# Oral health behaviors and associated factors in older adults: a cross-sectional study

**DOI:** 10.3389/froh.2025.1716964

**Published:** 2026-01-12

**Authors:** Inês Caetano-Santos, Giancarlo De la Torre Canales, Luís Proença, Mário Polido, José João Mendes, Helena Canhão, Ana Cristina Manso

**Affiliations:** 1 Egas Moniz Center for Interdisciplinary Research (CiiEM), Egas Moniz School of Health & Science, Almada, Portugal; 2 NOVA Medical School—Faculdade de Ciências Médicas, NOVA Medical School, Universidade Nova de Lisboa, Lisbon, Portugal; 3 Comprehensive Health Research Center (CHRC), NOVA Medical School, Universidade Nova de Lisboa, Lisbon, Portugal; 4 Division of Oral Diagnostics and Rehabilitation, Department of Dental Medicine, Karolinska Institutet, Huddinge, Sweden; 5 Department of Dentistry, Ingá University Center, Uningá, Paraná, Brazil

**Keywords:** older adults, oral health behaviors, oral hygiene, preventive dentistry, risk factors

## Abstract

**Background:**

Oral health behaviors play an essential role in maintaining oral health and preventing oral diseases, which are often neglected in older adults. This study aimed to investigate the prevalence of oral health behaviors and their associated factors in older adults.

**Methods:**

This cross-sectional study included participants aged 65 years or older who attended a university dental hospital in Portugal. Data were collected through a questionnaire on oral health behaviors, socioeconomic data, general health characteristics, self-perceived oral health and oral health-related quality of life. The oral clinical assessment included the Oral Hygiene Index-Simplified (OHI-S), the Decayed, Missing and Filled Teeth Index (DMFT), the Modified Community Periodontal Index, the presence of oral mucosal lesions, prosthetic presence, and prosthetic and treatment needs. Statistical analysis included descriptive and inferential methodologies. The association between potential risk indicators and oral health behaviors was assessed and modelled using logistic regression analysis.

**Results:**

A sample of 302 older adults participated in this study, and 56.0% were female. The mean age was 73.5 years (±5.8). Regarding oral health behaviors, 21.9% brushed less than twice a day, 52.1% did not use interdental devices and 49.3% last visited a dentist for treatment. Risk indicators for less frequent brushing were being male [OR = 3.00, 95% CI (1.65–5.47)], having a perceived prosthetic need [OR = 3.46, 95% CI (1.72–6.95)] and increased values of DMFT [OR = 1.15, 95% CI (1.08–1.22)]. Risk indicators for not using interdental devices were increased values of OHI-S [OR = 2.41, 95% CI (1.71–3.40)], being male [OR = 2.31, 95% CI (1.38–3.89)] and the presence of chronic diseases [OR = 1.87, 95% CI (1.03–3.39)]. Risk indicators for treatment being the reason for the last dental appointment were prosthetic presence [OR = 3.23, 95% CI (1.79–5.82)] and increased values of DMFT [OR = 1.06, 95% CI (1.01–1.11)].

**Conclusion:**

Assessment of oral health behaviors and associated factors should be considered to guide future public health strategies to improve compliance with oral health care among older adults and to prevent oral diseases.

## 1 Introduction

The aging of the global population has led to an increase in research on older adults (1–3). In terms of health, there has been evidence that this age group is more prone to chronic diseases and oral diseases, which are often correlated (4–6). Oral diseases can also cause pain, infections, reduced salivary flow, tooth loss, and changes in speech and chewing ability, affecting quality of life (5, 7). In addition, the increase in oral diseases among older adults has been linked to the fact that teeth remain in the oral cavity for longer, leading to an accumulation of oral diseases over the years, but also to the fact that this population finds it more difficult to maintain oral hygiene and visits the oral care services less often (3, 7, 8).

Effective oral health behaviors play an essential role in maintaining oral health and preventing oral diseases such as periodontal disease and dental caries, the main causes of tooth loss (9, 10). Toothbrushing is a simple and effective mechanical method of removing dental plaque, thereby reducing the risk of gingival inflammation and dental caries (10–12). Interdental cleaning further enhances plaque control in proximal areas that are not adequately reached by toothbrushing alone, contributing to the prevention of periodontal disease and interproximal caries (9, 11). Moreover, routine dental check-ups facilitate the early detection and timely management of oral diseases, which have been linked to a lower prevalence and severity of oral conditions (3, 8). Therefore, these preventive behaviors are essential and more cost-effective than restorative or rehabilitative treatments (9, 10).

Nevertheless, compliance with oral health behaviors may be influenced by limited oral health literacy, but also by loss of autonomy, dexterity and visual impairment, which may lead to some neglect of oral hygiene in terms of brushing, flossing, and oral care concerns (7, 13). In addition, a reduced number of visits to dental services could also be a consequence of low socioeconomic status, as these services are mainly provided in the private sector in some countries, such as Portugal, and due to the presence of chronic diseases leading to loss of mobility, or even social isolation. Moreover, regular dental visits have been associated with a lower prevalence of oral diseases than episodic visits (3, 8, 14, 15).

Socioeconomic status and general health indicators have already been associated with oral health care (4, 5, 8). However, most of the available evidence emerges from non-European countries whose healthcare systems and socioeconomic contexts may not be directly comparable to those in Portugal, where access to dental services varies significantly across population groups (14). In addition, several previous studies did not include measures of self-perception, which may also act as predisposing factors that influence motivation to seek oral health care, particularly among older adults (3, 16–18). Therefore, it is essential to explore these associations in Portuguese older adults by incorporating both objective and self-perceived indicators to better understand their influence on oral health care.

Knowing the oral health behaviors of a population and the factors associated with them allows to understand the reasons for non-adherence to these behaviors and to define public health strategies targeted at a specific population by planning oral health services and educating the population to improve their oral health and, consequently, their quality of life (17, 19). Notwithstanding, to the best of our knowledge, the literature on oral health behaviors in older adults is scarce, and it is poorly understood which indicators may influence compliance with oral health practices.

Therefore, the aim of this study was to investigate the prevalence of oral health behaviors and their association with socioeconomic factors, general health-related characteristics, oral health status, self-perception of oral health, and oral health-related quality of life among older adults.

## 2 Materials and methods

### 2.1 Study design and participants

This cross-sectional study included a convenience sample of 302 participants (*n* = 302) randomly selected from the Egas Moniz Dental Clinic, a university dental hospital in Almada, Portugal, between December 2020 and May 2022. Inclusion criteria included age of 65 years or older, not being institutionalized, being able to read, write, speak, and understand Portuguese, being able to comply with the study protocol, and not having disabilities such as blindness, deafness or dementia. All individuals who did not meet the inclusion criteria and those who refused to participate and sign the informed consent were excluded. This study followed the Strengthening Reporting of Observational Studies in Epidemiology (STROBE) guidelines (20).

### 2.2 Ethical considerations

This study was approved by the Ethics Committee of the Egas Moniz School of Health & Science (No. 896/2020) and by the Ethics Committee of the NOVA Medical School (No. 79/2020/CEFCM). The study protocol was conducted following the tenets of the Declaration of Helsinki, as revised in 2013. All participants were read and signed an informed consent form, accepting the conditions for participation in the study and the procedures involved. The data were collected anonymously by coding and were only intended for statistical processing and/or publication, maintaining the anonymity and confidentiality of the participants.

### 2.3 Data collection

Data were collected through a questionnaire and by oral clinical assessment at the Egas Moniz Dental Clinic. The self-reported questionnaire collected information on oral health behaviors, socioeconomic data, general health-related characteristics, self-perception of oral health and oral health-related quality of life. The oral clinical assessment included the Oral Hygiene Index-Simplified (OHI-S), the Decayed (D), Missing (M) and Filled (F) Teeth Index (DMFT), the Modified Community Periodontal Index (CPI), presence of oral mucosal lesions, prosthetic presence, and prosthetic and treatment needs.

#### 2.3.1 Oral health behaviors (dependent variables)

Oral hygiene behaviors included brushing frequency, which refers to brushing the teeth or, in the case of edentulous participants, cleaning the gums, oral mucosa, and tongue (<2x/day/≥2x/day), daily use of interdental devices, including dental floss or interdental brushes (yes/no) and last dental appointment reason (routine/treatment). From the total sample, 20 participants were excluded from the interdental device data collection because they were edentulous.

#### 2.3.2 Socioeconomic data

Socioeconomic data included sex (male/female), age, recorded as a continuous variable (years) and then converted into age groups (65–74/≥75), whether participants lived alone or not, educational level [elementary (≤4 years)/middle (5–12 years)/higher education (>12 years)] and monthly income (≤800 €/801–1,000 €/>1,000 €).

#### 2.3.3 General health-related characteristics

The presence of chronic diseases was classified as “yes” if the participant had a diagnosed chronic disease and “no” if had not, and medication was categorized as “yes” or “no” if the participant was taking medication or not.

Smoking habits were classified as “no” if not currently smoking, or “yes” if currently smoking. Alcohol intake was registered as a dichotomous variable (yes or no). For sugar intake, the question surveyed was “How often do you have meals containing sugar?” (“At least once a day or more/At least once a week/At least once a month or never”), explicitly stating that this question referred to the intentional consumption of foods or beverages with added sugars, including both meals and snacks (such as desserts, pastries, sweetened drinks). Self-reported height and weight were recorded, and body mass index (BMI, kg/m^2^) was calculated and categorized according to the nutritional status as <18.5 (underweight), 18.5–24.9 (healthy weight), ≥25 (overweight) (21).

#### 2.3.4 Self-perception of oral health

Self-perception of oral health was assessed through the following questions: “How would you describe your oral health?”, “How would you describe the condition of your teeth?” and “How would you describe the condition of your gums?”. The answers to the questions were classified as poor (“neither satisfied nor unsatisfied”, “unsatisfied” and “very unsatisfied”) or good (“very satisfied” and 'satisfied') (22). For xerostomia assessment, it was surveyed the question “Do you feel your mouth dry: often/occasionally/no?”. Perceived prosthetic needs and treatment needs were registered as dichotomous variables (need/no need).

#### 2.3.5 Oral health-related quality of life

Oral health-related quality of life (OHRQoL) was assessed using the validated Portuguese version of the Geriatric Oral Health Assessment Index (GOHAI) (23). The total score was the sum of the physical, psychosocial, and pain and discomfort domains.

#### 2.3.6 Oral health status

Clinical recordings were collected by a trained and calibrated general dentist who had previously undergone a calibration procedure on ten patients not included in the study. Measurement reliability and reproducibility were assessed by the intra-class correlation coefficient (ICC) and the intra-examiner agreement was 0.96. The room used for the observations had both natural and artificial lighting. The equipment used included an intraoral mirror, a CPI probe, gloves, a mask, and compresses (24).

The Oral Hygiene Index-Simplified (OHI-S), which is the sum of the Debris Index-Simplified (DI-S) and the Calculus Index-Simplified (CI-S), was calculated for each participant, excluding edentulous participants (25).

Dental status was recorded using DMFT index for each participant. Periodontal status was assessed using the CPI, which included the assessment of gingival bleeding (yes/no) and the measurement of periodontal pocket depth using a CPI probe. Loss of attachment was also measured in six index teeth: 17/16, 11, 26/27, 37/36, 31 and 46/47. From the total sample, 20 participants were excluded from the periodontal data collection because they were edentulous. For oral mucosal lesions assessment, an examination of oral mucosa and soft tissues was conducted and registered as presence (yes) or absence (no). These analyses were based on the World Health Organization's Oral Health Assessment Form for Adults (2013) (24).

Prosthetic presence was recorded if the participant could show or wear the prostheses (“yes”) or not (“no”). Prosthetic need was classified as “need” if the participant had missing functional teeth for which there was no existing prosthetic replacement (fixed or removable). Missing single teeth were only classified as “need” if they were in a functionally or aesthetically relevant position. “No need” was assigned if the participant had a prosthetic treatment or had all functional teeth. Missing teeth that would not normally require replacement, such as posterior teeth without functional impact or third molars, were not considered to be a prosthetic need. Treatment need was also assessed and registered as “need” (need dental treatment) or “no need” (routine/no treatment need).

### 2.4 Data analysis

The statistical analyses were performed using the software IBM SPSS Statistics v.29 and included descriptive and inferential methodologies. For descriptive analysis, categorical data were presented as frequency and percentage distributions, and numerical data as mean and standard deviation (SD). For the bivariate analysis phase, the Chi-square test and Fisher's exact test were used. The association between potential risk indicators and oral health behaviors was investigated and modelled using logistic regression analysis. Univariate models were used for the preliminary analysis. A multivariate model was then developed using the stepwise multivariate approach, based on variables that showed significance *p* ≤ 0.25 in the univariate model for each dependent variable. Wald statistics were used to assess the contribution of each variable to the model. For each variable examined, interactions were also considered. The final reduced models included risk indicators that were entered in steps: For oral hygiene frequency, DMFT (step 1), sex (step 2) and self-perceived prosthetic needs (step 3); the use of interdental devices included: OHI-S (step 1), sex (step 2) and chronic diseases (step 3), and for last appointment reason, prosthetic presence (step 1) and DMFT (step 2). Odds Ratio (OR) and 95% confidence intervals (95% CI) were calculated for both univariate and multivariate analyses. A significance level of 5% (*p* ≤ 0.05) was established in all inferential analyses.

## 3 Results

A descriptive data on socioeconomic, general health-related, and oral health behaviors of the study participants (*n* = 302) is presented in Table 1. Fifty-six per cent of the participants were female. The mean age was 73.5 years (±5.8). Half of the participants (50.0%) had only completed elementary school and 60.5% had a monthly income of ≤800 €. Regarding general health, 74.5% of the participants had been diagnosed with a chronic disease and 88.7% were taking medication. In terms of oral health behaviors, 21.9% brushed less than twice a day, 52.1% did not use an interdental device and 49.3% last visited a dentist for treatment.

**Table 1:** Descriptive data on socioeconomic status, general health and oral health behaviors of the study participants.

Variables	Categories	*n* (%)	Mean (SD)
Sex	Female	169 (56.0)	–
	Male	133 (44.0)	
Age group	65–74	187 (61.9)	73.5 (5.8)
	75+	115 (38.1)	
Living alone	Yes	99 (32.8)	–
	No	203 (67.2)	
Education level	Elementary	151 (50.0)	–
	Middle	114 (37.7)	
	Higher	37 (12.3)	
Monthly income (€)	≤800	178 (60.5)	–
	801–1,000	39 (13.3)	
	>1,000	77 (26.2)	
Chronic Diseases	Yes	225 (74.5)	–
	No	77 (25.5)	
Medication	Yes	268 (88.7)	–
	No	34 (11.3)	
Smoking habits	Smoker	25 (8.3)	–
	Non-smoker	277 (91.7)	
Alcohol intake	Yes	102 (33.8)	–
	No	200 (66.2)	
Sugar intake	Never/At least once a month	128 (42.4)	–
	At least once a week	47 (15.6)	–
	At least once a day or more	127 (42.1)	–
BMI (kg/m^2^)	Underweight	1 (0.3)	–
	Healthy weight	109 (36.1)	
	Overweight	192 (63.6)	
Oral hygiene frequency	<2x/day	66 (21.9)	–
	≥2x/day	236 (78.1)	
Use of interdental devices	No	147 (52.1)	–
	Yes	135 (47.9)	
Last appointment reason	Treatment	149 (49.3)	–
	Routine	153 (50.7)	

BMI, body mass index; SD, standard deviation.

Data on self-perceived oral health and OHRQoL are shown in Table 2. Most participants perceived their oral health as “good” (78.7%), although 80.5% reported a need for treatment. The mean total GOHAI score was 31.4 (±4.3).

**Table 2:** Descriptive data on self-perceived oral health, and OHRQoL of the study participants.

Variables	Categories	*n* (%)	Mean (SD)
Self-perception (oral health)	Poor	64 (21.3)	–
	Good	237 (78.7)	
Self-perception (teeth)	Poor	77 (27.6)	–
	Good	202 (72.4)	
Self-perception (gums)	Poor	39 (12.9)	–
	Good	263 (87.1)	
Xerostomia	Often	80 (26.5)	–
	Occasionally	87 (28.8)	
	No	135 (44.7)	
Perceived prosthetic need	Need	50 (16.6)	–
	No need	252 (83.4)	
Perceived treatment need	Need	243 (80.5)	–
	No need	59 (19.5)	
GOHAI (total score)	–	–	31.4 (4.3)
GOHAI (physical function)	–	–	10.2 (2.3)
GOHAI (psychosocial function)	–	–	7.5 (1.1)
GOHAI (pain and discomfort)	–	–	13.8 (1.7)

GOHAI, geriatric oral health assessment index; SD, standard deviation.

Table 3 shows the oral clinical characteristics of the participants. The mean DMFT was 23.2 (±6.2) and the mean OHI-S was 1.1 (±0.8). More than half of the participants had gingival bleeding (56.8%) and 46.4% had a 4–5 mm periodontal pocket. In terms of prosthetic rehabilitation, 69.2% were wearing dentures, but 25.8% of participants needed dentures. Overall, most participants were considered to require dental treatment.

**Table 3:** Descriptive data on oral clinical status of the study participants.

Variables	Categories	*n* (%)	Mean (SD)
Decayed teeth	–	–	3.2 (3.1)
Missing teeth	–	–	16.1 (7.1)
Filled teeth	–	–	3.2 (3.3)
DMFT	–	–	23.2 (6.2)
DI-S	–	–	0.7 (0.5)
CI-S	–	–	0.4 (0.5)
OHI-S	–	–	1.1 (0.8)
Gingival bleeding	No	121 (43.2)	–
	Yes	159 (56.8)	
Periodontal pocket	No pocket (0–3 mm)	81 (28.9)	–
	4–5 mm	130 (46.4)	
	≥6 mm	69 (24.6)	
Loss of attachment	0–3 mm	82 (29.8)	–
	4–5 mm	137 (49.8)	
	6–8 mm	51 (18.5)	
	9–11 mm	4 (1.5)	
	≥12 mm	1 (0.4)	
Oral mucosal lesions	No	275 (91.1)	–
	Yes	27 (8.9)	
Prosthetic presence	Dentures	209 (69.2)	–
	No dentures	93 (30.8)	
Prosthetic need	Need	78 (25.8)	–
	No need	224 (74.2)	
Treatment need	Need	251 (83.1)	–
	No need	51 (16.9)	

CI-S, calculus index-simplified; DI-S, debris index-simplified; DMFT, decayed, missing, filled teeth index; OHI-S, oral hygiene index-simplified; SD, standard deviation.

Data on socioeconomic status, general health, self-perceived oral health, OHRQoL and clinical status according to oral health behaviors are shown in Table 4. Regarding brushing frequency, sex and education were associated with this practice, with male sex (*n* = 42, 62.3%; *p* < 0.001) and with participants with null or elementary school (*n* = 40, 60.6%; *p* = 0.045) brushing less often. Smoking habits and alcohol intake also showed an association (*p* = 0.022 and *p* = 0.010, respectively). In terms of clinical oral status, participants with higher mean scores for missing teeth (*p* < 0.001), DMFT (*p* < 0.001), OHI-S (*p* = 0.001) and with prosthetic needs (*p* = 0.027) reported brushing less often.

**Table 4:** Socioeconomic status, general health, self-perceived oral health, OHRQoL and clinical status according to oral health behaviors.

Variables	Categories	Brushing frequency (*N* = 302)		*p*-value	Use of interdental devices (*N* = 282)		*p*-value	Last appointment reason (*N* = 302)		*p*-value
		<2x/day	≥2x/day		No	Yes		Treatment	Routine	
Sex	Female	24 (36.4)	145 (61.4)	**<0**.**001**	67 (45.6)	92 (68.1)	**<0**.**001**	82 (55.0)	87 (56.9)	0.749
	Male	42 (63.6)	91 (38.6)		80 (54.4)	43 (31.9)		67 (45.0)	66 (43.1)	
Age group	65–74	39 (59.1)	148 (62.7)	0.592	85 (57.8)	88 (65.2)	0.205	90 (60.4)	97 (63.4)	0.592
	75+	27 (40.9)	88 (37.3)		62 (42.2)	47 (34.8)		59 (39.6)	56 (36.6)	
Living alone	Yes	19 (28.8)	80 (33.9)	0.434	46 (31.3)	44 (32.6)	0.815	49 (32.9)	50 (32.7)	0.97
	No	47 (71.2)	156 (66.1)		101 (68.7)	91 (67.4)		100 (67.1)	103 (67.3)	
Education level	Null/Elementary	40 (60.6)	111 (47.0)	**0**.**045**	84 (57.1)	57 (42.2)	**0**.**027**	82 (55.0)	69 (45.1)	0.223
	Middle	23 (34.8)	91 (38.6)		49 (33.3)	55 (40.7)		51 (34.2)	63 (41.2)	
	Higher	3 (4.5)	34 (14.4)		14 (9.5)	23 (17.0)		16 (10.7)	21 (13.7)	
Monthly income (€)	≤800	44 (69.8)	134 (58.0)	0.230	94 (65.7)	70 (53.0)	**0**.**034**	89 (61.0)	89 (60.1)	0.532
	801–1,000	6 (9.5)	33 (14.3)		20 (14.0)	17 (12.9)		22 (15.1)	17 (11.5)	
	>1,000	13 (20.6)	64 (27.7)		29 (20.3)	45 (34.1)		35 (24.0)	42 (28.4)	
Chronic Diseases	Yes	49 (74.2)	176 (74.6)	0.956	118 (80.3)	91 (67.4)	**0**.**014**	113 (75.8)	112 (73.2)	0.599
	No	17 (25.8)	60 (25.4)		29 (19.7)	44 (32.6)		36 (24.2)	41 (26.8)	
Medication	Yes	60 (90.9)	208 (88.1)	0.529	132 (89.8)	120 (88.9)	0.805	135 (90.6)	133 (86.9)	0.312
	No	6 (9.1)	28 (11.9)		15 (10.2)	15 (11.1)		14 (9.4)	20 (13.1)	
Smoking habits	Smoker	10 (15.2)	15 (6.4)	**0**.**022**	11 (7.5)	11 (8.1)	0.835	12 (8.1)	13 (8.5)	0.889
	Non-smoker	56 (84.8)	221 (93.6)		136 (92.5)	124 (91.9)		137 (91.9)	140 (91.5)	
Alcohol intake	Yes	31 (47.0)	71 (30.1)	**0**.**01**	65 (38.9)	37 (27.4)	**0**.**035**	57 (38.3)	45 (29.4)	0.104
	No	35 (53.0)	165 (69.9)		102 (61.1)	98 (72.6)		92 (61.7)	108 (70.6)	
Sugar intake	Never/At least once a month	25 (37.9)	103 (43.6)	0.314	64 (38.3)	64 (47.4)	0.233	61 (40.9)	67 (43.8)	0.879
	At least once a week	8 (12.1)	39 (16.5)		26 (15.6)	21 (15.6)		24 (16.1)	23 (15.0)	
	At least once a day or more	33 (50.0)	94 (39.8)		77 (46.1)	50 (37.0)		64 (43.0)	63 (41.2)	
BMI (kg/m^2^)	Underweight/healthy weight	26 (39.4)	84 (35.6)	0.571	51 (34.7)	50 (37.0)	0.682	56 (37.6)	54 (35.3)	0.679
	Overweight	40 (60.6)	152 (64.4)		96 (65.3)	85 (63.0)		93 (62.4)	99 (64.7)	
Self-perception (oral health)	Poor	22 (33.3)	42 (17.9)	**0**.**007**	44 (26.5)	20 (14.8)	**0**.**014**	32 (21.6)	32 (20.9)	0.881
	Good	44 (66.7)	193 (82.1)		122 (73.5)	115 (85.2)		116 (78.4)	121 (79.1)	
Self-perception (teeth)	Poor	27 (46.6)	50 (22.6)	**<0**.**001**	50 (34.5)	27 (20.1)	**0**.**007**	39 (29.3)	38 (26.0)	0.538
	Good	31 (53.4)	171 (77.4)		95 (65.5)	107 (79.9)		94 (70.7)	108 (74.0)	
Self-perception (gums)	Poor	9 (13.6)	30 (12.7)	0.843	23 (13.8)	16 (11.9)	0.621	20 (13.4)	19 (12.4)	0.795
	Good	57 (86.4)	206 (87.3)		144 (86.2)	119 (88.1)		129 (86.6)	134 (87.6)	
Xerostomia	Often	19 (28.8)	61 (25.8)	0.279	42	38	0.322	42 (28.2)	38 (24.8)	0.698
	Occasionally	23 (34.8)	64 (27.1)		54	33		40 (26.8)	47 (30.7)	
	No	24 (36.4)	111 (47.0)		71	64		67 (45.0)	68 (44.4)	
Perceived prosthetic need	Need	21 (31.8)	29 (12.3)	**<0**.**001**	33 (19.8)	17 (12.6)	0.096	20 (13.4)	30 (19.6)	0.148
	No need	45 (68.2)	207 (87.7)		134 (80.2)	118 (87.4)		129 (86.6)	123 (80.4)	
Perceived treatment need	Need	53 (80.3)	190 (80.5)	0.970	136 (81.4)	107 (79.3)	0.635	113 (75.8)	130 (85.0)	**0**.**045**
	No need	13 (19.7)	46 (19.5)		31 (18.6)	28 (20.7)		36 (24.2)	23 (15.0)	
GOHAI (total score)	–	31.0 (4.1)	31.5 (4.3)	0.295	31.2 (4.1)	31.7 (4.5)	0.202	30.8 (4.1)	32.0 (4.4)	**<0**.**001**
GOHAI (physical function)	–	9.9 (2.3)	10.3 (2.3)	0.092	10.0 (2.4)	10.6 (2.1)	**0**.**04**	9.6 (2.5)	10.8 (2.0)	**<0**.**001**
GOHAI (psychosocial function)	–	7.4 (1.2)	7.5 (1.0)	0.49	7.3 (1.1)	7.6 (1.0)	**0**.**027**	7.6 (1.2)	7.5 (1.0)	0.127
GOHAI (pain and discomfort)	–	13.7 (1.8)	13.8 (1.6)	0.736	13.8 (1.8)	13.8 (1.5)	0.208	13.6 (1.7)	14.0 (1.6)	0.056
Decayed teeth	–	3.7 (3.8)	3.2 (2.9)	0.930	3.2 (3.2)	3.2 (3.0)	0.638	2.3 (2.8)	3.7 (3.3)	**0**.**003**
Missing teeth	–	18.3 (7.2)	15.3 (6.8)	**<0**.**001**	18.2 (7.1)	13.9 (6.4)	**<0**.**001**	18.8 (6.8)	13.7 (6.4)	**<0**.**001**
Filled teeth	–	2.7 (3.2)	3.3 (3.4)	0.075	2.0 (2.7)	4.4 (3.5)	**<0**.**001**	2.6 (3.0)	3.7 (3.5)	**0**.**003**
DMFT	–	24.7 (6.1)	21.8 (5.7)	**<0**.**001**	23.4 (6.0)	21.6 (5.7)	**0**.**006**	24.8 (5.7)	21.6 (6.2)	**<0**.**001**
OHI-S	–	1.4 (0.8)	1.0 (0.8)	**0**.**001**	1.4 (0.7)	0.8 (0.5)	**<0**.**001**	1.2 (0.9)	1.0 (0.8)	**0**.**047**
Gingival bleeding	–	0.56 (0.50)	0.61 (0.49)	0.850	0.63 (0.48)	0.51 (0.50)	0.108	0.50 (0.50)	0.63 (0.48)	**0**.**022**
Periodontal pocket	–	0.95 (0.73)	1.00 (0.70)	0.763	0.99 (0.71)	0.93 (0.74)	0.591	1.00 (0.74)	0.93 (0.71)	0.454
Loss of attachment	–	0.94 (0.76)	0.91 (0.73)	0.864	0.99 (0.77)	0.88 (0.74)	0.387	0.91 (0.83)	0.95 (0.68)	0.319
Oral mucosal lesions	Yes	7 (10.6)	20 (8.5)	0.592	12 (7.2)	15 (11.1)	0.235	17 (11.4)	10 (6.5)	0.138
	No	59 (89.4)	216 (91.5)		155 (92.8)	120 (88.9)		132 (88.6)	143 (93.5)	
Prosthetic presence	Dentures	40 (60.6)	169 (71.6)	0.087	98 (66.7)	93 (68.9)	0.690	125 (83.9)	84 (54.9)	**<0**.**001**
	No dentures	26 (39.4)	67 (28.4)		49 (33.3)	42 (31.1)		24 (16.1)	69 (45.1)	
Prosthetic need	Need	24 (36.4)	54 (22.9)	**0**.**027**	44 (29.9)	32 (23.7)	0.239	22 (14.8)	56 (36.6)	**<0**.**001**
	No need	42 (63.6)	182 (77.1)		103 (70.1)	103 (76.3)		127 (85.2)	97 (63.4)	
Treatment need	Need	56 (84.8)	195 (82.6)	0.670	136 (81.4)	115 (85.2)	0.387	124 (83.2)	127 (83.0)	0.960
	No need	10 (15.2)	41 (17.4)		31 (18.6)	20 (14.8)		25 (16.8)	26 (17.0)	

BMI, body mass index; DMFT, decayed, missing, filled teeth index; GOHAI, geriatric oral health assessment index; N, number of participants; OHI-S, oral hygiene index—simplified; statistical analysis for a significance level *p* < 0.05. *p*-values in bold indicate a statistically significant association.

Regarding the use of interdental devices, sex (*p* < 0.001) and education level (*p* = 0.027) were associated with this behavior. In addition, most participants with a monthly income of ≤800€ (*n* = 94, 65.7%; *p* = 0.034) reported not using interdental devices. The presence of chronic diseases (*p* = 0.014) and alcohol consumption (*p* = 0.035) were also significantly associated with the use of interdental devices. In terms of self-perception and OHRQoL, self-perception of oral health (*p* = 0.014) and teeth (*p* = 0.007), and the physical (*p* = 0.040) and psychosocial (*p* = 0.027) domains of the GOHAI also showed a significant association. Clinical indicators significantly associated with the use of interdental devices were missing teeth (*p* < 0.001), filled teeth (*p* < 0.001), DMFT (*p* = 0.006), and OHI-S (*p* < 0.001) (Table 4).

Regarding the reason for the last dental visit, we did not find a significant association with socioeconomic and general health characteristics. However, participants with perceived treatment need (*n* = 113, 75.8%; *p* = 0.045) had more treatment appointments as the last dental visit. In addition, participants with lower means in GOHAI [30.8 (±4.1)] and its physical domain [9.6 (±2.5)] showed having more treatment appointments (*p* < 0.001). Finally, some oral health indicators were significantly associated with the reason for the last dental appointment, including DMFT (*p* < 0.001), OHI-S (*p* = 0.047), gingival bleeding (*p* = 0.022), prosthetic presence (*p* < 0.001) and prosthetic need (*p* < 0.001) (Table 4).

Table 5 shows the multivariate logistic regression model of the risk indicators for brushing frequency. Participants with a higher mean DMFT (OR = 1.15, *p* < 0.001) and male participants (OR = 3.00, *p* < 0.001) were more likely to brush less than twice a day. Participants with a perceived need for dentures were also more likely to brush less often (OR = 3.46, *p* < 0.001).

**Table 5:** Multivariate logistic regression analysis (final reduced model*) towards the outcome “brushing frequency <2x/day”.

Variable		OR (95% CI)	*p*
DMFT		1.15 (1.08–1.22)	<0.001
Sex	Female	1	–
	Male	3.00 (1.65–5.47)	<0.001
Perceived prosthetic need	No need	1	–
	Need	3.46 (1.72–6.95)	<0.001

* The model was statistically significant, *χ*^2^(3) = 44.023, *p* < 0.001, explained 20.9% (Nagelkerke *R*^2^) of the variance, and correctly classified 79.8% of cases. CI, confidence interval; DMFT, decayed, missing, filled teeth index; OR, odds ratio.

The risk indicators for the use of interdental devices are shown in Table 6. Participants with a higher mean OHI-S were more likely not to use an interdental device (OR = 2.41, *p* < 0.001). Similar results were found for male participants (OR = 2.31, *p* = 0.002). Participants with diagnosed chronic diseases were also more likely not to use an interdental device (OR = 1.87, *p* = 0.039).

**Table 6:** Multivariate logistic regression analysis (final reduced model*) towards the outcome “no use of interdental device”.

Variable		OR (95% CI)	*p*
OHI-S		2.41 (1.71–3.40)	<0.001
Sex	Female	1	–
	Male	2.31 (1.38–3.89)	0.002
Chronic Diseases	No	1	–
	Yes	1.87 (1.03–3.39)	0.039

* The model was statistically significant, *χ*^2^(3) = 50.406, *p* < 0.001, explained 21.8% (Nagelkerke *R*^2^) of the variance, and correctly classified 66.7% of cases. CI, confidence interval; OHI-S, oral hygiene index-simplified; OR, odds ratio.

Table 7 presents the multivariate logistic regression model of the risk indicators for the last appointment reason being treatment. Participants with prosthetic treatment had more than two times more likelihood of having a treatment appointment (OR = 3.23, *p* < 0.001). In addition, participants with a higher mean DMFT were more likely to have treatment appointments (OR = 1.06, *p* = 0.021).

**Table 7:** Multivariate logistic regression analysis (final reduced model*) towards the outcome “last appointment reason—treatment”.

Variable		OR (95% CI)	*p*
Prosthetic presence	No dentures	1	–
	Dentures	3.23 (1.79–5.82)	<0.001
DMFT		1.06 (1.01–1.11)	0.021

* The model was statistically significant, *χ*^2^(2) = 33.063, *p* < 0.001, explained 14.9% (Nagelkerke *R*^2^) of the variance, and correctly classified 65.4% of cases. CI, confidence interval; DMFT, decayed, missing, filled teeth index; OR, odds ratio.

## 4 Discussion

To the best of our knowledge, few studies have specifically examined oral health behaviors and their associated factors among Portuguese older adults, and available evidence in this population remains limited. In our study, although brushing frequency was relatively high, the use of interdental cleaning devices and participation in routine preventive dental care were considerably lower. Among the significant associated indicators, sex, perceived prosthetic need, and DMFT emerged as the most relevant predictors of brushing frequency. Similarly, sex, the presence of chronic diseases, and OHI-S were predictors of interdental cleaning practices, while DMFT and prosthetic presence were associated with the reason for the last dental appointment.

Oral health behaviors play an important role in the prevention of oral diseases, which are particularly prevalent in older adults since they retain their natural teeth longer (2, 7, 9). Brushing frequency, the use of interdental devices and the reason for the last dental appointment were the oral health behaviors assessed in this study, as these are indicators considered essential for the prevention of oral diseases (11, 12, 26). Our results showed that the great majority of participants brushed their teeth at least twice a day, which is consistent with other national studies (27–29) and better than in other countries (26, 30). This result confirms that toothbrushing is a well-established and simple mechanical method of removing plaque, which is considered essential for maintaining oral health and preventing periodontal disease and dental caries, the main causes of tooth loss (10, 11). Notwithstanding, we found that male participants were more likely to brush less often. In this regard, several studies have shown that oral health is more closely associated with women's perception of quality of life and even their perception that oral diseases can be painful and embarrassing (18, 30, 31). In addition, participants with a self-perceived need for prosthetic treatment may not give much importance to their oral health and may not be aware of the impact of oral health on their general health, namely proper chewing and nutrition, which is also reflected in their oral health behaviors (2). Additionally, patients with higher DMFT may be less motivated in their oral hygiene behaviors or may have pain or discomfort from oral conditions that lead them to avoid brushing their teeth (19, 32).

In addition to brushing, other devices such as dental floss, brushes, stickers, and irrigators are also recommended for oral hygiene. Interdental cleaning is associated with less coronal and interproximal caries, less periodontal disease, and fewer missing teeth (9). Our findings showed that approximately half of the participants used interdental devices. Although this result is not ideal, it is better than most national and international studies (9, 11, 27–29). This could be explained by the fact that dental floss and interdental brushes were analyzed together, or by the fact that these participants had voluntarily come to this dental hospital for their dental appointments and were, therefore, more motivated in their oral hygiene behavior. The use of interdental brushes has been described as the most effective method of interdental plaque removal due to higher patient acceptance and ease of use (33). In contrast, patient compliance with daily flossing is low, mainly due to a lack of motivation and difficulties in using dental floss, especially in this age group (9, 33). Furthermore, not using an interdental device was shown to be more associated with male participants, which is consistent with the previously described behavior of less frequent brushing and consistent with other studies (9, 30, 34, 35). In addition, older adults who have a higher prevalence of chronic diseases, some of which can lead to a decline in cognitive and fine motor skills, are more likely to have poor dexterity and inadequate interdental oral hygiene. This can lead to a lack of motivation to perform this action more thoroughly (15, 36, 37). Finally, poor oral hygiene indices may be related to a general lack of motivation to engage in more meticulous oral hygiene practices (9, 26, 38).

Regular dental check-ups are one of the most important measures to maintain oral health (11, 12). However, several studies have shown that the adult population tends to seek oral health care only in an emergency and perceives routine visits as less important and financially inaccessible, a tendency that worsens with age (11, 27). The main reasons for older adults neglecting routine appointments are related to low energy, perceived inconvenience, fear and lack of access to dental care (37, 39). In addition, the oral health care system in Portugal is mostly provided by private clinics, increasing financial constraints on regular dental visits (28). In this study, denture wearers were more likely to attend treatment appointments as their last dental visit, possibly due to an increase in the number of denture-related appointments, such as repairs and adjustments, since these were the third most common reasons for the last dental visit in Portugal in 2023, according to the oral health report of the Portuguese Dental Association (29). Besides, an increased number of decayed, filled or missing teeth may lead to more visits to the dentist for treatment or maintenance of dental restorations or prosthetic treatment. For these participants, routine visits where oral health education and professional support for oral behavioral techniques can be provided are less frequent, so they may have poorer oral health care (26).

Contrary to other studies, age and socioeconomic status were not found to be major risk indicators for poorer oral health behaviors in the present study, which may be related to the fact that all participants in this study were older and of a similar age. In addition, oral hygiene tools appear to be affordable for this population, but the cost of oral health services may be a greater challenge for individuals with lower socioeconomic status, leading to an accumulation of oral diseases (12, 18, 34).

Although it has been associated with poorer oral health in other studies, most of the indicators of self-perceived oral health were not associated with oral health care variables in the final models of this study, except self-perceived prosthetic need (22, 40–42). However, indicators of self-perceived oral health and quality of life are particularly relevant to study in the older population, as lack of awareness of the importance of oral health has been shown to negatively impact on demand for oral health services (43).

Our study has some limitations that should be considered. Firstly, this is a cross-sectional study therefore, any cause-and-effect test is hindered. Further, clinical trials or longitudinal studies that monitor changes over time may extend the findings of this study and allow a more comprehensive exploration of this topic, potentially incorporating additional variables such as oral health literacy. Secondly, the sample size adopted was a convenience sample for the period of the study, so representativeness may be limited. Furthermore, individuals who attend university dental hospitals often differ from the wider Portuguese older population in terms of socioeconomic background, health-seeking behaviors, and level of motivation for oral health care. University dental hospitals may attract patients who are more motivated to seek treatment, benefit from reduced-cost services, or require complex care, potentially introducing socioeconomic and behavioral biases. As such, the results may not fully reflect the oral health behaviors or needs of older adults who do not regularly seek dental care or who rely exclusively on private dental services. Therefore, caution should be used when generalizing these findings to the entire Portuguese older population. Self-reported information may also be subjected to information bias, such as recall bias, false reporting affected by social desirability, or undiagnosed diseases. Residual confounding from unknown or unmeasured factors was possible, although the final models were carefully adjusted for identified risk indicators. Therefore, the results of this study should be analyzed with caution and cannot be generalized to the entire Portuguese older population.

As a final remark, the results of this cross-sectional study are useful for a comprehensive analysis of oral health behaviors in older adults and highlight the main risk indicators for poorer oral health care in this population. In addition, understanding predictive indicators of poor oral health care is essential to improve the approach of oral health professionals and to develop tailored educational strategies. This study also underlines the importance of ongoing strategies developed in the National Oral Health Plan, improving access to oral health care in public services, hospitals and health centers, and increasing preventive dentistry to avoid oral diseases.

## 5 Conclusions

Our findings show that older adults have good compliance with brushing frequency, but still lower adherence to interdental cleaning and to routine dental appointments. Factors associated with poor oral health behaviors were sex (male), increased DMFT values, perceived prosthetic need, having chronic diseases, increased OHI-S values and prosthetic presence.

## Data Availability

The raw data supporting the conclusions of this article will be made available by the authors, without undue reservation.

## References

[1] United Nations. World Population Ageing 2019—Highlights. (2019). Available online at: https://www.un.org/en/development/desa/population/publications/pdf/ageing/WorldPopulationAgeing2019-Report.pdf (Accessed July 30, 2024).

[2] Patel J Wallace J Doshi M Gadanya M Ben Yahya I Roseman J Oral health for healthy ageing. Lancet Healthy Longev. (2021) 2(8):e521–7. 10.1016/S2666-7568(21)00142-2 36098001

[3] Ortíz-Barrios LB Granados-García V Cruz-Hervert P Moreno-Tamayo K Heredia-Ponce E Sánchez-García S. The impact of poor oral health on the oral health-related quality of life (OHRQoL) in older adults: the oral health status through a latent class analysis. BMC Oral Health. (2019) 19(1):141. 10.1186/s12903-019-0840-3 31291933 PMC6622000

[4] Petersen PE Bourgeois D Ogawa H Estupinan-Day S Ndiaye C. The global burden of oral diseases and risks to oral health. Bull World Health Organ. (2005) 83(9):661–9. 16211157 PMC2626328

[5] Gil-Montoya JA de Mello AL Barrios R Gonzalez-Moles MA Bravo M. Oral health in the elderly patient and its impact on general well-being: a nonsystematic review. Clin Interv Aging. (2015) 10:461–7. 10.2147/CIA.S54630 25709420 PMC4334280

[6] Lauritano D Moreo G Della Vella F Di Stasio D Carinci F Lucchese A Oral health status and need for oral care in an aging population: a systematic review. Int J Environ Res Public Health. (2019) 16(22):4558. 10.3390/ijerph16224558 31752149 PMC6888624

[7] Tuuliainen E Nihtilä A Komulainen K Nykänen I Hartikainen S Tiihonen M The association of frailty with oral cleaning habits and oral hygiene among elderly home care clients. Scand J Caring Sci. (2020) 34(4):938–47. 10.1111/scs.12801 31845365

[8] Yellowitz JA Schneiderman MT. Elder’s oral health crisis. J Evid Based Dent Pract. (2014) 14:191–200. 10.1016/j.jebdp.2014.04.011 24929604

[9] Worthington HV MacDonald L Poklepovic Pericic T Sambunjak D Johnson TM Imai P Home use of interdental cleaning devices, in addition to toothbrushing, for preventing and controlling periodontal diseases and dental caries. Cochrane Database Syst Rev. (2019) 4(4):CD012018. 10.1002/14651858.CD012018.pub2 30968949 PMC6953268

[10] Yaacob M Worthington HV Deacon SA Deery C Walmsley AD Robinson PG Powered versus manual toothbrushing for oral health. Cochrane Database Syst Rev. (2014) 6:CD002281. 10.1002/14651858.CD002281.pub3 PMC7133541 24934383

[11] Tadin A Badrov M. Oral health knowledge, self-assessed oral health behavior, and oral hygiene practices among the adult general population in Croatia. Healthcare. (2023) 12(1):88. 10.3390/healthcare12010088 38200994 PMC10778950

[12] An R Li S Li Q Luo Y Wu Z Liu M Oral health behaviours and oral health-related quality of life among dental patients in China: a cross-sectional study. Patient Prefer Adherence. (2022) 16:3045–58. 10.2147/PPA.S385386 36387048 PMC9651070

[13] Lamster IB Asadourian L Del Carmen T Friedman PK. The aging mouth: differentiating normal aging from disease. Periodontol 2000. (2016) 72(1):96–107. 10.1111/prd.12131 27501493

[14] Simões J Augusto GF do Céu A Ferreira MC Jordão M Calado R Ten years since the 2008 introduction of dental vouchers in the Portuguese NHS. Health Policy. (2018) 122(8):803–7. 10.1016/j.healthpol.2018.07.013 30054096

[15] Marchesan JT Byrd KM Moss K Preisser JS Morelli T Zandona AF Flossing is associated with improved oral health in older adults. J Dent Res. (2020) 99(9):1047–53. 10.1177/0022034520916151 32321349 PMC7375740

[16] Salih MA Ali RW Nasir EF. Linking the demographic, socio-economic and oral health status to oral health-related quality of life of the Sudanese older adults: a cross sectional study. BMC Oral Health. (2023) 23(1):371. 10.1186/s12903-023-03089-6 37291534 PMC10251693

[17] Guevara-Canales JO Morales-Vadillo R Sacsaquispe-Contreras SJ Alberca-Ramos DE Morgenstern-Orezzoli H Cava-Vergiú CE. Association between self-perceived oral health and clinical indicators. Oral Health Prev Dent. (2018) 16(1):33–41. 10.3290/j.ohpd.a39685 29335684

[18] Olusile AO Adeniyi AA Orebanjo O. Self-rated oral health status, oral health service utilization, and oral hygiene practices among adult Nigerians. BMC Oral Health. (2014) 14:140. 10.1186/1472-6831-14-140 25427860 PMC4258058

[19] Salih MA Ali RW Nasir EF. Oral health status and associated factors among Sudanese older adults: a cross-sectional study. Gerodontology. (2022) 39(4):408–17. 10.1111/ger.12611 34859486

[20] von Elm E Altman DG Egger M Pocock SJ Gøtzsche PC Vandenbroucke JP Strengthening the reporting of observational studies in epidemiology (STROBE) statement: guidelines for reporting observational studies. Br Med J. (2007) 335(7624):806–8. 10.1136/bmj.39335.541782.AD 17947786 PMC2034723

[21] Weir CB Jan A. BMI Classification percentile and cut off points. In: StatPearls. Treasure Island (FL): StatPearls Publishing (2024). p. 1–4. 10.1371/journal.pone.0337951

[22] Fagundes MLB do Amaral Júnior OL Menegazzo GR do Nascimento Tôrres LH. Factors associated with self-perceived oral health in different age groups. Community Dent Oral Epidemiol. (2022) 50(6):476–83. 10.1111/cdoe.12673 34176140

[23] Carvalho C Manso AC Escoval A Salvado F Nunes C. Translation and validation of the Portuguese version of geriatric oral health assessment Index (GOHAI). RPSP. (2013) 31(2):153–9. 10.1016/j.rpsp.2013.10.002

[24] World Health Organization. Oral health surveys: Basic methods (5th ed). (2013). Available online at: https://iris.who.int/bitstream/handle/10665/97035/9789241548649_eng.pdf?sequence=1 (Accessed July 30, 2024).

[25] Greene JC Vermillion JR. The simplified oral hygiene index. J Am Dent Assoc. (1964) 68:7–13. 10.14219/jada.archive.1964.0034 14076341

[26] Konstantopoulou K Kossioni AE. Association between oral hygiene information sources and daily dental and denture care practices in urban community-dwelling older adults. J Clin Med. (2023) 12(8):2881. 10.3390/jcm12082881 37109220 PMC10142920

[27] Melo P Marques S Silva OM. Portuguese self-reported oral-hygiene habits and oral status. Int Dent J. (2017) 67(3):139–47. 10.1111/idj.12273 27981568 PMC9376680

[28] Guerreiro E Botelho J Machado V Proença L Mendes JJ Manso AC. Caries experience and risk indicators in a Portuguese population: a cross-sectional study. Int J Environ Res Public Health. (2023) 20(3):2511. 10.3390/ijerph20032511 36767876 PMC9915840

[29] Ordem dos Médicos Dentistas: Barómetro de Saúde Oral 2023. (2023). Available online at: https://www.omd.pt/content/uploads/2023/11/VIII-Barómetro-Nacional-de-Saúde-Oral_2023.pdf (Accessed July 30, 2024).

[30] Shamsoddin E. Dental floss as an adjuvant of the toothbrush helps gingival health. Evid Based Dent. (2022) 23(3):94–6. 10.1038/s41432-022-0818-x 36151277

[31] Su S Lipsky MS Licari FW Hung M. Comparing oral health behaviours of men and women in the United States. J Dent. (2022) 122:104157. 10.1016/j.jdent.2022.104157 35545161

[32] Moradi G Mohamadi Bolbanabad A Moinafshar A Adabi H Sharafi M Zareie B. Evaluation of oral health status based on the decayed, missing and filled teeth (DMFT) Index. Iran J Public Health. (2019) 48(11):2050–7. 31970104 PMC6961190

[33] Ng E Lim LP. An overview of different interdental cleaning aids and their effectiveness. Dent J. (2019) 7(2):56. 10.3390/dj7020056 PMC6630384 31159354

[34] Fleming EB Nguyen D Afful J Carroll MD Woods PD. Prevalence of daily flossing among adults by selected risk factors for periodontal disease-United States, 2011–2014. J Periodontol. (2018) 89(8):933–9. 10.1002/JPER.17-0572 29644699 PMC6434526

[35] Liang L Aris IM. Minimal changes in daily flossing behavior among US adults from 2009 through 2020. J Am Dent Assoc. (2024) 155(7):587–596.e2. 10.1016/j.adaj.2024.04.001 38752966

[36] Viebranz S Dederichs M Kwetkat A Schüler IM. Effectiveness of individual oral health care training in hospitalized inpatients in geriatric wards. Int J Environ Res Public Health. (2023) 20(5):4275. 10.3390/ijerph20054275 36901286 PMC10001549

[37] Bakker MH de Smit MJ Valentijn A Visser A. Oral health assessment in institutionalized elderly: a scoping review. BMC Oral Health. (2024) 24(1):272. 10.1186/s12903-024-04025-y 38402181 PMC10893687

[38] Kim SJ Lee JY Kim SH Cho HJ. Effect of interdental cleaning devices on proximal caries. Community Dent Oral Epidemiol. (2022) 50(5):414–20. 10.1111/cdoe.12690 34369614

[39] Niesten D van Mourik K van der Sanden W. The impact of frailty on oral care behavior of older people: a qualitative study. BMC Oral Health. (2013) 13(61). Available online at: https://pmc.ncbi.nlm.nih.gov/articles/PMC3819177/pdf/1472-6831-13-61.pdf 24175989 10.1186/1472-6831-13-61 PMC3819177

[40] Krause L Seeling S Starker A. Self-perceived oral health and associated factors among adults in Germany. Results from GEDA 2019/2020-EHIS. Bundesgesundheitsblatt Gesundheitsforschung Gesundheitsschutz. (2021) 64(8):967–76. 10.1007/s00103-021-03376-z 34232335 PMC8316182

[41] Shariati M Noori N Hoseinzadeh M Feizi M Kazemian A. Adults’ estimated prevalence of healthy behavior in society and self-reported oral health status and behaviours. Community Dent Health. (2023) 40(3):182–6. 10.1922/CDH_00089Shariati05 37549184

[42] Nascimento M Cunha Soares F Dahllöf G Burgos Souto Maior G Kvist T Colares V. Determinants of self-perceived oral health in adolescents: a cross-sectional study. Int J Paediatr Dent. (2021) 31(2):254–61. 10.1111/ipd.12664 32419168

[43] Piuvezam G de Lima KC. Self-perceived oral health status in institutionalized elderly in Brazil. Arch Gerontol Geriatr. (2012) 55(1):5–11. 10.1016/j.archger.2011.04.017 21621283

